# Visualization of chemical space for medicinal chemists

**DOI:** 10.1186/1758-2946-6-S1-O4

**Published:** 2014-03-11

**Authors:** Peter Ertl

**Affiliations:** 1 Novartis Institutes for BioMedical Research, CH-4056 Basel, Switzerland

## 

One of the most common tasks that cheminformatics experts in pharmaceutical industry are facing practically daily is analysis and visualization of large collections of molecules. Typical areas, where this is needed are analysis and enhancement of company compound archive, analysis of high-throughput screening data, design of combinatorial libraries, chemogenomics analyses and many others. But also researchers in academia are facing similar challenges when analyzing large public molecular databases that become available recently or even structures generated *in silico*. This presentation will provide overview of various methods used to analyze and visualize chemical space with particular focus on needs of medicinal chemists.

When displaying results, for chemists it is of great importance that the molecules are represented by their actual structures, or at least by their scaffolds and not only by points as it is common in other scientific fields. This particular requirement makes chemistry visualizations challenging because of necessity to squeeze a lot of information on rather limited computer screen real estate.

In the presentation various chemistry visualization techniques will be discussed, starting from classical display of molecules as tables and grids, through visualization based on analysis of scaffold, up to advanced cheminformatics visualizations techniques recently developed at Novartis, such as a method for natural ordering or scaffolds or Molecule Cloud diagrams.

